# The Multifunctional Sorting Protein PACS-2 Controls Mitophagosome Formation in Human Vascular Smooth Muscle Cells through Mitochondria-ER Contact Sites

**DOI:** 10.3390/cells8060638

**Published:** 2019-06-25

**Authors:** Manon Moulis, Elisa Grousset, Julien Faccini, Kevin Richetin, Gary Thomas, Cecile Vindis

**Affiliations:** 1 INSERM, UMR 1048, Institute of Metabolic and Cardiovascular Diseases/I2MC, F-31342 Toulouse, France; manon.moulis@uclouvain.be (M.M.); elisa.grousset@inserm.fr (E.G.); julien.faccini@free.fr (J.F.); 2 University of Toulouse III, F-31342 Toulouse, France; 3 Center for Psychiatric Neuroscience, Department of Psychiatry, Lausanne University Hospital, 1005 Lausanne, Switzerland; kevin.richetin@chuv.ch; 4 Department of Microbiology and Molecular Genetics, University of Pittsburgh School of Medicine, Pittsburgh, PA 15219, USA; thomasg@pitt.edu; 5 Hillman Cancer Center, University of Pittsburgh School of Medicine, Pittsburgh, PA 15213, USA

**Keywords:** PACS-2, mitochondria-associated ER membranes (MAMs), mitochondria, mitophagy, apoptosis, atherosclerosis, vascular smooth muscle cell

## Abstract

Mitochondria-associated ER membranes (MAMs) are crucial for lipid transport and synthesis, calcium exchange, and mitochondrial functions, and they also act as signaling platforms. These contact sites also play a critical role in the decision between autophagy and apoptosis with far reaching implications for cell fate. Vascular smooth muscle cell (VSMC) apoptosis accelerates atherogenesis and the progression of advanced lesions, leading to atherosclerotic plaque vulnerability and medial degeneration. Though the successful autophagy of damaged mitochondria promotes VSMC survival against pro-apoptotic atherogenic stressors, it is unknown whether MAMs are involved in VSMC mitophagy processes. Here, we investigated the role of the multifunctional MAM protein phosphofurin acidic cluster sorting protein 2 (PACS-2) in regulating VSMC survival following a challenge by atherogenic lipids. Using high-resolution confocal microscopy and proximity ligation assays, we found an increase in MAM contacts as in PACS-2-associated MAMs upon stimulation with atherogenic lipids. Correspondingly, the disruption of MAM contacts by PACS-2 knockdown impaired mitophagosome formation and mitophagy, thus potentiating VSMC apoptosis. In conclusion, our data shed new light on the significance of the MAM modulatory protein PACS-2 in vascular cell physiopathology and suggest MAMs may be a new target to modulate VSMC fate and favor atherosclerotic plaque stability.

## 1. Introduction

The close membrane appositions between the mitochondrion and the endoplasmic reticulum (ER) form 10–50 nm wide contact sites commonly referred as mitochondria-associated ER membranes (MAMs) or mitochondria-ER contacts (MERCs) [1]. Such contact sites support the inter-organelle communication involved in the detection of extracellular inputs and stressful situations. MAMs have been described in species ranging from yeast to mammals [2]. Several studies identified that MAMs are crucial for processes including lipid transport and synthesis, calcium exchange, mitochondrial functions, and apoptosis [3,4,5]. A large number of ER and mitochondria-associated proteins have been identified in MAMs and described to participate as molecular tethers (including Ca^2+^ ion channels) such as inositol 1,4,5-trisphosphate receptor type 1 (IP3R1) and voltage-dependent anion channel 1 (VDAC1); chaperones like the glucose-regulated protein 75 (Grp75); proteins involved in the regulation of mitochondrial shape and dynamic, including dynamin-related protein 1 (DRP1) and mitofusin 2 (MFN2); and the multifunctional sorting protein, phosphofurin acidic cluster sorting protein 2 (PACS-2) [6].

The involvement of MAMs in the critical balance between cell survival and death has been recently suggested, particularly because these contact sites play a vital role in modulating the decision between autophagy and apoptosis. For instance, the anti-apoptotic protein Bcl-2 binds to the autophagy protein Beclin-1 on the ER membrane. The Bcl-2-Beclin-1 complex can regulate the switch between apoptotic and autophagic pathways [4]. In addition, the early autophagosomal marker, ATG5, localizes to punctae on mitochondria and are followed by the late autophagosomal marker, LC3 [7,8]. Likewise, the pre-autophagosome/autophagosome marker ATG14 complex translocates to an ER-based early autophagy membrane assembly zone associated with MAMs where it is maintained, in part, via MFN2 and PACS-2 [7,8]. Indeed, PACS-2 controls the apposition of mitochondria with the ER, as depletion of PACS-2 causes BAP31-dependent mitochondria fragmentation and uncoupling from the ER [9]. Moreover, PACS-2 has been shown to control cell fate by translocating Bid to mitochondria [9] or by promoting ataxia telengectasia (ATM) to drive nuclear factor kappa B (NF-κB) activation and the induction of anti-apoptotic Bcl-xL [10]. PACS-2 was also identified as an essential TNF-related apoptosis-inducing ligand (TRAIL) effector, one required for killing tumor cells in vitro and virally infected hepatocytes in vivo [11].

Vascular smooth muscle cells (VSMCs) control multiple attributes of vessel homeostasis, including contraction, dilation, and vessel remodeling. Consequently, VSMCs are a major cellular determinant of arterial wall disease. In atherosclerosis, VSMCs are the first to carry lipid retention and lipid overload through foam cell formation and death. Because VSMCs contribute to the synthesis of matrix components and form the fibrous cap of atherosclerotic plaques, the induction of VSMC apoptosis increases plaque vulnerability and medial degeneration, leading to plaque thrombogenicity [12]. However, the mechanisms controlling plaque stability versus plaque rupture are complex, and the oxidizing and inflammatory environment generated by the presence of pro-atherogenic factors (low-density lipoproteins (LDL) and oxidized lipids, oxidative stress, cytokines) can trigger either pro-survival or pro-death processes which are concomitantly activated in cells [13].

The induction of autophagy plays an important role in the response of VSMCs to various atherogenic stressors, such as oxidized lipids [14]. Though autophagy is often considered to be a non-selective degradative pathway [15], the selective autophagy of damaged organelles is critical for the maintenance of cellular homeostasis. Indeed, damaged mitochondria can also be discarded by a specific form of autophagy known as mitophagy [16]. Mitophagy takes place in specific developmental processes, such as the maturation of erythrocytes [17], and it also follows pathological mitochondrial damage in order to eliminate dysfunctional mitochondria and prevent cell death [18]. Alterations in mitochondrial morphology (fragmentation and remodeling) and function (decline in mitochondrial membrane potential and increase in radical production) are observed during the early stages of apoptosis. These events are known to be prerequisites for the initiation of mitophagy [19,20], which promotes cell survival by preventing the release of pro-apoptotic factors into the cytosol and the activation of downstream cell death pathways [21].

The indication that mitophagy occurs at MAM contacts was first shown in yeast where the ER-mitochondria encounter structure (ERMES) complex, which tethers the ER and mitochondria, is colocalized with the site of mitophagosome biogenesis [22]. We previously reported the importance of mitophagy in VSMC fate upon apoptotic stress [23]. However, the relevance of MAM formation and function in the regulation of VSMC mitophagy and cell death is unknown. Here, we report that the tether-inducing protein PACS-2 accumulates at mitochondria-ER contact sites in human VSMCs challenged with oxidized low-density lipoproteins (LDL) and thereby increases the MAM contacts. The disruption of MAM contacts by PACS-2 deletion impairs mitophagosome formation and mitophagy, thus potentiating VSMC apoptosis. These findings suggest that MAM-associated PACS-2 is a critical regulator of human VSMCs fate during oxidized LDL-induced mitophagy.

## 2. Materials and Methods

### 2.1. Reagents and Antibodies

Oligomycin (O4876), carbonyl cyanide 4-(trifluoromethoxy) phenylhydrazone (FCCP) (C2920), antimycin A (A8674), rotenone (R8875), carbonyl cyanide 3-chlorophenylhydrazone (CCCP) (C2759), bafilomycin A1 (B1793), sodium pyruvate (P5280), and glucose (G7021) were purchased from Sigma-Aldrich (St. Quentin Fallavier, France). MitoTracker Deep Red (M22426) and l-glutamine (25030-024) were purchased from ThermoFisher Scientific (Illkirch, France). Cyto-ID (ENZ-51031) was purchased from Enzo Life Sciences (Villeurbanne, France). Anti-Mitofusin 2 (rabbit, 9482), anti-Grp75 (rabbit, 3593), anti-LC3B (rabbit, 2775), and anti-Myc (mouse, 2276) were from Cell Signaling Technology (Saint Quentin Yvelines, France). Anti-Tom20 (rabbit, sc-11415), anti-Tom20-AF488 (rabbit, sc-17764), anti-Parkin (mouse, sc-32282), and anti-Ero1-Lα (mouse, sc-365526) were from Santa Cruz Biotechnology (Heidelberg, Germany). Anti-VDAC1 (mouse, ab14734), anti-Parkin (rabbit, ab15954), and anti-Beclin-1 (mouse, ab114071) were from Abcam (Paris, France). Anti-KDEL (mouse, ADI-SPA-827-D) was from Enzo Life Sciences. Anti-PACS-2 (rabbit, 19508-1-AP) was from Proteintech (Manchester, UK), and anti-PACS-2 (rabbit, clone 18143) was previously reported [9]. Anti-IP3R1 (rabbit, 07-1213) was from Merck Millipore (Molsheim, France). Anti-β-actin (mouse, A2228) and anti-P62 (rabbit, P0067) were from Sigma-Aldrich.

### 2.2. Cell Culture and Transfection

Human primary VSMCs were obtained from the mesenteric arteries of postmortem organ donors in agreement with the French “Agence de Biomédecine” and the ethics committee of the University Hospital of Toulouse, France. Briefly, the arteries were cut longitudinally, and small pieces of the media were carefully stripped from the vessel wall and cultured until VSMCs migrated from the explants (1–2 weeks) and could be passed (3 weeks after the first appearance of cells) [24]. The VSMC phenotype was verified with a specific α-smooth muscle actin staining. The primary cultured human VSMCs were then used to generate an immortalized cell line by using a SV40T antigen [23]. SV40T and contractile phenotype markers-expressing human VSMC were maintained in a Dulbecco’s modified Eagle’s medium (DMEM, 61965-026, ThermoFisher Scientific, Illkirch, France) supplemented with 10% fetal calf serum, penicillin (100 U/mL), and streptomycin (10 µg/mL) at 37 °C under a humidified atmosphere with 5% CO_2_. Human VSMCs were used between passage 7 and 17 for all experiments, and they were cultured in DMEM without serum before each experiment.

Mouse embryonic fibroblasts (MEFs) deleted for the PACS-2 protein and isolated from PACS-2^−/−^ mice were kindly provided by Professor Gary Thomas (Department of Microbiology and Molecular Genetics, University of Pittsburg, Pittsburg, USA) and cultivated as human VSMC in complete DMEM with serum and antibiotics [11].

For siRNA transfection, ON-TARGET plus Human PACS-2 (L-022015-01), ON-TARGET plus Human Mfn2 (L-012961-00), and ON-TARGET plus Human PRKN (L-003603-00) from Dharmacon (Compiegne, France) were, respectively, directed against PACS-2, MFN2, and Parkin, which were then transfected (200–400 nM) in an Opti-MEM medium (31985-047, ThermoFisher Scientific) using the Hiperfect reagent (301705, Qiagen, Hilden, Germany) according to the manufacturer’s instructions.

For plasmids transfection, the pcDNA3-PACS-2 plasmid was kindly provided by Professor Gary Thomas (Department of Microbiology and Molecular Genetics, University of Pittsburg, Pittsburg, USA), and the SIN-PGK-MitoTimer plasmid was kindly provided by Dr. Kevin Richetin (Lausanne University Hospital, Lausanne, Switzerland). Cells were transiently transfected (0.5 µg/mL) in a DMEM serum-free medium using the GeneCellin reagent (GC1000, Eurobio, Les Ulis, France) according to the manufacturer’s instructions.

### 2.3. LDL Isolation and Oxidation

LDL from normal human pooled sera were prepared by ultracentrifugation and dialyzed against phosphate buffered saline (PBS) containing 100 µM ethylenediaminetetraacetic acid (EDTA). The LDL pool was then diluted to 2 g/L with PBS into a final volume of 3 mL. LDL were mildly oxidized by UV-C for 2 h in the presence of 5 µM CuSO_4_, as previously reported [25]. Oxidized LDL contained 4.2–7.4 nmoles of TBARS (thiobarbituric acid-reactive substances)/µg ApoB. Relative electrophoretic mobility (REM) and 2,4,6-trinitrobenzenesulfonic acid (TNBS) reactive amino groups were 1.2–1.3 times and 85–92% of native LDL, respectively.

### 2.4. Cell Immunofluorescence: Classic and High-Resolution Stimulated Emission Depletion (STED) Confocal Microscopy

After the treatments, cells grown on glass coverslips were fixed in PBS-4% paraformaldehyde (PFA) for 10 min at room temperature (RT) and then washed and permeabilized with PBS-0.1% TritonX100 for 10 min at RT. After blocking with PBS containing 3% bovine serum albumin (BSA) for 45 min at RT, cells were incubated with the indicated primary antibodies (1/100) for 1 h at RT and revealed with Alexa Fluor-conjugated secondary antibodies (1/1000) for 1 h at RT. Nuclei were labeled with 4′,6-diamidino-2-phenylindole (DAPI) and cells were mounted with a fluorescent mounting medium (DAKO, 3023). For classic confocal microscopy, Alexa Fluor goat anti-mouse (A11001), anti-rabbit (A11008) 488, goat anti-mouse (A11003), anti-rabbit (A11010) 546, and anti-rabbit (A21244) 647 from ThermoFisher Scientific were used, and images were acquired on a Zeiss LSM 780 inverted fluorescence confocal microscope. For high-resolution stimulated emission depletion (STED) microscopy, the same immunocytochemistry protocol was used, but the cells were grown on thinner glass coverslips, primary antibodies were incubated at 1/50, coverslips were mounted with Mowiol and F(ab’)2 goat anti-rabbit 594 (A11072, ThermoFisher Scientific, Illkirch, France), and goat anti-mouse Abberior STAR RED (52283, Sigma-Aldrich, St. Quentin Fallavier, France) were used as specific secondary antibodies. Images were acquired on a Leica SP8 inverted fluorescence confocal microscope equipped with a STED module. Between 8 and 20 images of one cell were acquired (63×, zoom 3 or 5) per condition for each experiment, and all images were analyzed using Image J software. The colocalization analysis between the different channels was realized by measuring both Pearson’s colocalization coefficient (perinuclear cell area) before binarization and objects colocalization area after binarization. For this latter measure, images were first thresholded and binarized for each channel. Then, the objects area for each channel, as their area fraction that colocalizes with objects of another channel, were obtained with Set Measurements and Analyse Particules tabs on Image J software. These values of area fraction were obtained for each object of each channel and corresponded to the percentage of each object area in one channel colocalized with objects in the other channel. For the colocalization measure between three channels, a colocalized binary mask of two channels was first created. Then, the corresponding absolute area value of colocalization was calculated for each object in an excel file, and the total sum of these area values per image were finally used to measure the parameters of colocalization between channels.

### 2.5. Duolink^®^ In Situ Proximity Ligation Assay

Duolink^®^ in situ proximity ligation assay (PLA) is a powerful technique allowing for the detection, visualization, and quantification of protein–protein interactions (<40 nm) as individual fluorescent dots by microscopy. This technology combines immunodetection and molecular biology techniques based on antibodies affinity and the amplification of oligonucleotidic sequences coupled to secondary antibodies. After the treatments, cells were fixed and permeabilized as described for cell immunofluorescence. Blocking (1 h at RT) and incubation with primary (1/100, 1 h at RT) and secondary antibodies (1/5, 1 h at 37 °C) using anti-rabbit PLUS (DUO92002) and anti-mouse MINUS (DUO92004) kits, followed by ligation and amplification steps using the detection reagents red kit (DUO92008), were performed according to the manufacturer’s protocol (Sigma-Aldrich, St. Quentin Fallavier, France). Coverslips were mounted with a mounting medium containing DAPI (DUO82040) and fixed with varnish. Fluorescence was analyzed on a Zeiss LSM 780 inverted fluorescence confocal microscope, 5–10 fields of about 15 cells per condition for each experiment were acquired, and the number of interactions per cell was then analyzed using Image J software.

### 2.6. Western-Blot Analysis

After the treatments, cells were washed in cold PBS, and proteins were extracted in a solubilizing buffer (10 mM Tris pH 7.4, 150 mM NaCl, 1% Triton X-100, 1% sodium deoxycholate, 0.1% sodium dodecyl sulfate, 1 mM sodium orthovanadate, 1 mM sodium pyrophosphate, 5 mM sodium fluoride, 1 mM phenylmethylsulfonyl fluoride, 1 μg/mL leupeptin, 1 μg/mL aprotinin) for 30 min on ice. For western-blot analyses, 30 μg of protein cell extracts were resolved by SDS-polyacrylamide gel electrophoresis and transferred onto polyvinylidene fluoride (PVDF) membranes (Immobilon, IPVH 00010, Merck Millipore, Molsheim, France). Subsequently, membranes were probed with the indicated primary antibodies and developed with the secondary antibodies coupled to horseradish peroxidase using an ECL chemoluminescence kit (RPN21016, Amersham, Pittsburgh, PA, USA) and a Chemidoc Touch system (Bio-Rad, Marnes-la-Coquette, France). Membranes were stripped and reprobed with an anti-β-actin antibody to control the equal loading of proteins. The quantification of the protein bands was performed using Image Lab software (Image Lab 6.0.1, Bio-Rad, Marnes-la-Coquette, France).

### 2.7. Mitochondrial Complexes I and III Activity Assay

The specific activity of complexes I and III was measured as rotenone-sensitive NADH cytochrome c reductase activity according to Spinazzi and coll [26]. After treatment, cells plated in 24-well plates were scraped and centrifuged in cold PBS containing proteases inhibitors. The cell pellet was resuspended in a mannitol buffer with pH 7.2 (mannitol 225 mM, saccharose 75 mM, Tris HCl 10 mM, EDTA 0.1 mM), frozen in liquid nitrogen, and kept at −80 °C. After protein content determination for each condition, 40 µL of cellular homogenate (0.2 µg/mL final concentration) was added to 1940 µL of a reaction medium (potassium phosphate buffer pH 7.5 50 mM, BSA 1 mg/mL, cytochrome c 100 µM, KCN 1 mM). Five µL rotenone (2.5 mM) was added to 900 µL of a reaction medium and incubated at 37 °C for 5 min before the reaction was started by adding 100 µL NADH (2 mM), and 550 nm absorbance was read every 15 s for 3 min. The specific enzymatic activity was then calculated from the Beer–Lambert law using the molar extinction coefficient of cytochrome c (18.5 L/mmol/cm) and expressed in nmol/min/mg of protein.

### 2.8. Mitochondrial Bioenergetics Analysis

Mitochondrial respiration was determined using a Seahorse XF24e extracellular flux analyzer (Seahorse Bioscience, Agilent Technologies France, Les Ulis, France) as per the manufacturer’s instructions and as described in [27]. Briefly, human VSMCs were plated into XF24 plates (100777-004, Seahorse Bioscience) at 40,000 cells per well in a DMEM medium and were allowed to adhere for 24 h. The basal oxygen consumption rate (OCR) was determined, and then oligomycin (1 μM), FCCP (1 μM) and antimycin A/rotenone (1 μM) were sequentially added to measure basal respiration, maximal respiration, ATP production and spare respiratory capacity. For normalization, cells were lysed in the XF24 plates using a protein lysis buffer (50 µL/well), and protein concentration was determined using the Bradford method.

### 2.9. Confocal Imaging of MitoTimer and Ratiometric Analysis

Cells grown on glass coverslips were transiently transfected with MitoTimer plasmid in a DMEM serum free medium using the GeneCellin reagent (GC1000, Eurobio, Les Ulis, France) according to the manufacturer’s instructions. After treatments, cells were fixed, the coverslips were mounted on glass slides, and images were acquired by confocal microscopy (Zeiss LSM780, Zeiss, Marly le Roi, France) for both green (excitation/emission 483/500 nm) and red (excitation/emission 558/583 nm) channels [28]. Images were then analyzed with Image J software; green and red channels were merged as a single binarized image to detect all mitochondria, and a mask was created from it to measure the mean fluorescence intensity for each mitochondrion, both in the green and red channels. A measurement of the red/green ratio then allowed us to determine the oxidative status of mitochondria (ratiometric image). For the assessment of mitochondria and PACS-2 colocalization, a pixel colocalization was performed, and mitochondria were then classified according to their colocalization with PACS-2 as their red/green ratio level.

### 2.10. Mitophagy Flux Assessment Using Flow Cytometry

Cells plated into 6-well plates were stained in a medium with 10 nM of MitoTracker Deep Red (MitoTR) (M22426, Invitrogen) for 15 min at 37 °C, washed and trypsinized for 5 min at 37 °C, and then resuspended in PBS. Using an LSRFortessa flow cytometer (Becton Dickinson, Franklin Lakes, NJ, USA) 20,000 cells were acquired (FACSDiva software, BD FACSDiva v8.0.1, Becton Dickinson), and the data were analyzed using the single cell analysis software FlowJo. The mean fluorescence (FL4 channel) in the viable cell population was plotted and normalized against that of untreated cells as described in [29]. Mitophagy flux compares the MitoTR levels with and without lysosomal inhibitors and is calculated as the ratio of MitoTR fluorescence in the presence of lysosomal inhibitors to that in the absence of inhibitors, normalized to the corresponding value in control cells.

### 2.11. Annexin V/propidium Iodide and FACS Analyses

After oxidized LDL stimulation, cells were collected, resuspended, and stained with dyes of the Annexin V FITC Detection Kit (Eurobio, ABC500FI, Les Ulis, France) according to the manufacturer’s instructions and as described in [23]. Cells were then analyzed using a FACSVerse flow cytometer (Becton Dickinson). The percentage of Annexin V positive (and propidium iodide positive or negative) cells was determined and compared between the different conditions.

### 2.12. Statistical Analyses

Results are expressed as the mean ± SEM. The data normality distribution was assessed using the Shapiro–Wilk normality test. Parametric tests were realized when the data assumed the normal distribution assumption if not non-parametric tests were applied. The differences between two groups were analyzed with unpaired Student’s t-test, Wilcoxon or Mann–Whitney tests. Differences between more than two groups were analyzed by the Kruskal–Wallis test and followed by Dunn’s post-hoc test or two-way ANOVA, followed by Tukey’s or Holm–Sidak’s post-hoc tests if there was a significant interaction between groups. All statistical analyses were performed using GraphPad Prism software (v 6.01, GraphPad Software, San Diego, CA, USA).

## 3. Results

### 3.1. The Sorting Protein PACS-2 Accumulates at Mitochondria-ER Contact Sites in Response to Oxidized LDL in Human VSMCs

**Figure 1:**
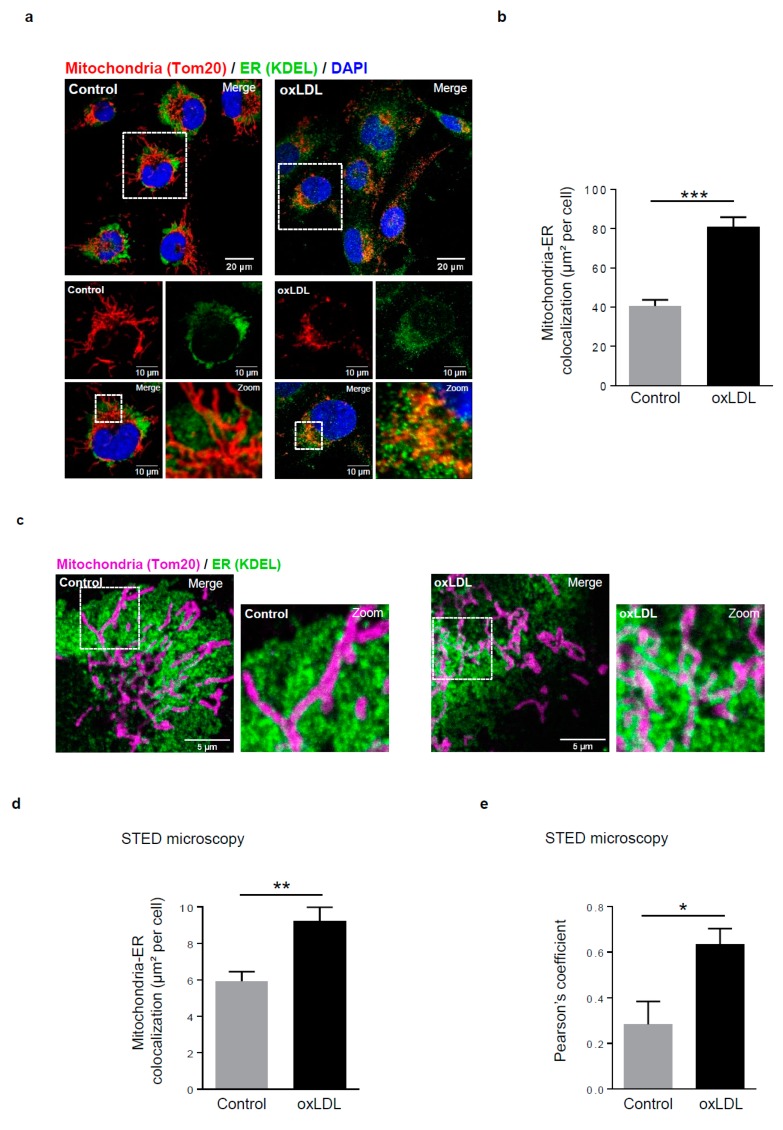
Mitochondria-ER contact sites are increased in response to oxidized low-density lipoproteins (LDL) stimulation in human vascular smooth muscle cells (hVSMCs). (**a**) Representative images of mitochondria (Tom20, red) and ER (KDEL, green) contacts in hVSMCs at baseline conditions (Control) or stimulated with oxidized LDL (oxLDL, 200 µg ApoB/mL, 5 h). Images were obtained with an LSM 780 confocal microscope, scale bar 20 µm, zoom scale bar 10 µm. (**b**) Analysis of the colocalization area between mitochondria and ER using Image J software. The graph represents the mean ± SEM of 10 cells analyzed per experiment for each condition (*n* = 3; Student’s *t* test, *** *p* < 0.001). (**c**) Representative images of mitochondria (Tom20, magenta) and ER (KDEL, green) contacts in hVSMCs at baseline conditions (Control) or stimulated with oxidized LDL (oxLDL, 200 µg ApoB/mL, 5 h). Images were obtained with the high-resolutive stimulated emission depletion (STED) technology on a SP8 confocal microscope, scale bar 5 µm. (**d**) The colocalization area between mitochondria and ER and (**e**) the Pearson’s colocalization coefficient were measured with Image J software. The graphs represent the mean ± SEM of 8 cells analyzed per experiment for each condition (*n* = 3; Student’s t and Mann–Whitney tests, * *p* < 0.05, ** *p* < 0.01).

Firstly, we sought to examine whether the number of mitochondria-ER contacts in human VSMCs (hVSMCs) are increased upon cell stimulation with oxidized lipids (oxidized low-density lipoproteins, oxidized LDL). The colocalization area between mitochondria labelled with the translocase of outer membrane 20 (Tom20) and the ER marker KDEL was measured by confocal microscopy and showed a two-fold significant increase compared to the baseline condition (Figure 1a,b). For higher resolution analysis, the mitochondria-ER contacts were measured by high-resolution STED confocal microscopy, which allows for at least a 20 nm lateral resolution and a 40–50 nm axial resolution in comparison to the 200 nm lateral resolution limit of classical confocal microscopy (Figure 1c). A significant increase of the mitochondria-ER contacts upon oxidized LDL treatment was still detected (Figure 1d). The calculation of the Pearson’s correlation coefficient (0.28 versus 0.63) confirmed that there is a significant association between mitochondria and the ER in oxidized LDL-stimulated hVSMCs compared to baseline conditions (Figure 1e).

**Figure 2:**
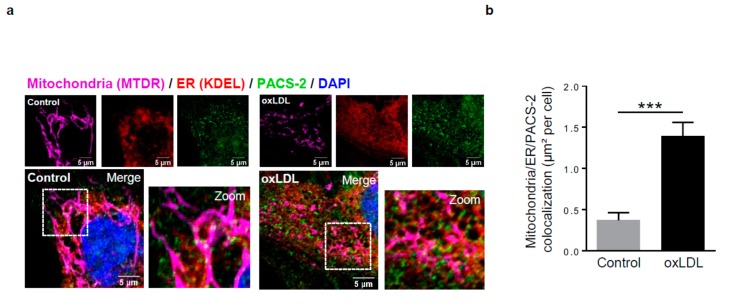

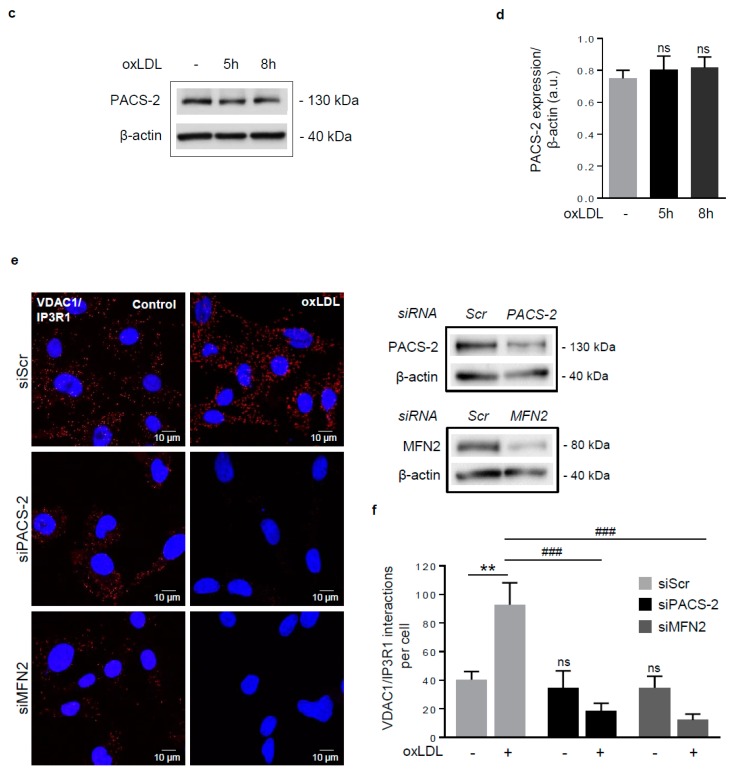
Phosphofurin acidic cluster sorting protein 2 (PACS-2) accumulates at mitochondria-ER contact sites in response to oxidized LDL in hVSMCs and is required for mitochondria-associated ER membranes (MAM) interaction. (**a**) Representative images of mitochondria (Mitotracker Deep Red, MTDR, magenta), ER (KDEL, red) and PACS-2 (green) in hVSMCs at baseline conditions (Control) or stimulated with oxidized LDL (oxLDL, 200 µg ApoB/mL, 5 h). Images were obtained with an LSM 780 confocal microscope, scale bar 5 µm. (**b**) Analysis of the colocalization area between mitochondria, ER, and PACS-2 using Image J software. The graph represents the mean ± SEM of 10 cells analyzed per experiment for each condition (*n* = 3, Mann–Whitney test, *** *p* < 0.001). (**c**) Western-blot analysis of PACS-2 time course expression in hVSMCs stimulated with oxidized LDL (oxLDL, 200 µg ApoB/mL). (**d**) The graph represents the densitometric analysis of the expression level of the PACS-2 protein. The data are expressed as mean ± SEM of four independent experiments (one-way ANOVA test, ns, non-significant). (**e**) (right panel), western-blot analysis of PACS-2 and mitofusin 2 (MFN2) expression levels in hVSMCs after siRNA transfection (scr, scrambled); (left panel), Representative images of the interactions between two MAM proteins, voltage-dependent anion channel 1 (VDAC1) and inositol 1,4,5-trisphosphate receptor type 1 (IP3R1) (red dots), obtained by a proximity ligation assay (PLA). hVSMCs were transfected with scrambled siRNA (siScr), PACS-2 siRNA (siPACS-2) or MFN2 siRNA (siMFN2) and stimulated or not with oxidized LDL (oxLDL, 200 µg ApoB/mL, 5 h). Images were obtained with an LSM 780 confocal microscope. (**f**) The number of VDAC1/IP3R1 interactions per cell was analyzed using Image J software, the graph represents the mean ± SEM of 10 wide field images per experiment for each condition (*n* = 3; two-way ANOVA with treatment (*) and siRNA (#) as category factors and Tukey’s post-hoc test, ** *p* < 0.01, ^###^ *p* < 0.001, ns, non-significant).

The sorting protein PACS-2 is involved not only in the tethering between mitochondria and the ER but also in the control of cell fate at mitochondria-ER contact sites [9,30]. However, the potential role of PACS-2 as a check point for MAM formation in hVSMCs, as well as for their cell fate, has not been reported. We first checked the expression of PACS-2 at MAM sites in hVSMCs. Triple-color imaging demonstrated the MAM localization of PACS-2 in hVSMCs (Figure 2a) in addition to its significant accumulation at MAM sites under oxidized LDL stimulation (Figure 2b). Moreover, PACS-2 accumulation at MAM sites was independent to an increase of its protein expression level (Figure 2c,d). We further assessed the requirement of PACS-2 for the oxidized LDL-induced changes in interacting MAM proteins by using the in situ PLA, a recently developed method [31] allowing for the visualization and quantification of protein–protein interactions ranging from 0 to 40 nm through dual antibody recognition. For the interactions between VDAC1 and IP3R1 (Figure 2e) or VDAC1 and Grp75 (Figure S1a), three organelle-surface proteins at the MAM interface were detected as intracellular fluorescent red dots. The number of PLA dots per cell was increased in oxidized LDL stimulated-hVSMCs but significantly prevented after PACS-2 silencing (Figure 2e–f, Figure S1a,b). The specificity of the assay was also demonstrated by the inhibition of VDAC1/IP3R1 and VDAC1/Grp75 interactions after MFN2 silencing (Figure 2e–f, Figure S1a,b).

### 3.2. Dysfunctional Mitochondria Induce the Formation of Mitochondria-ER Contact Sites

**Figure 3:**
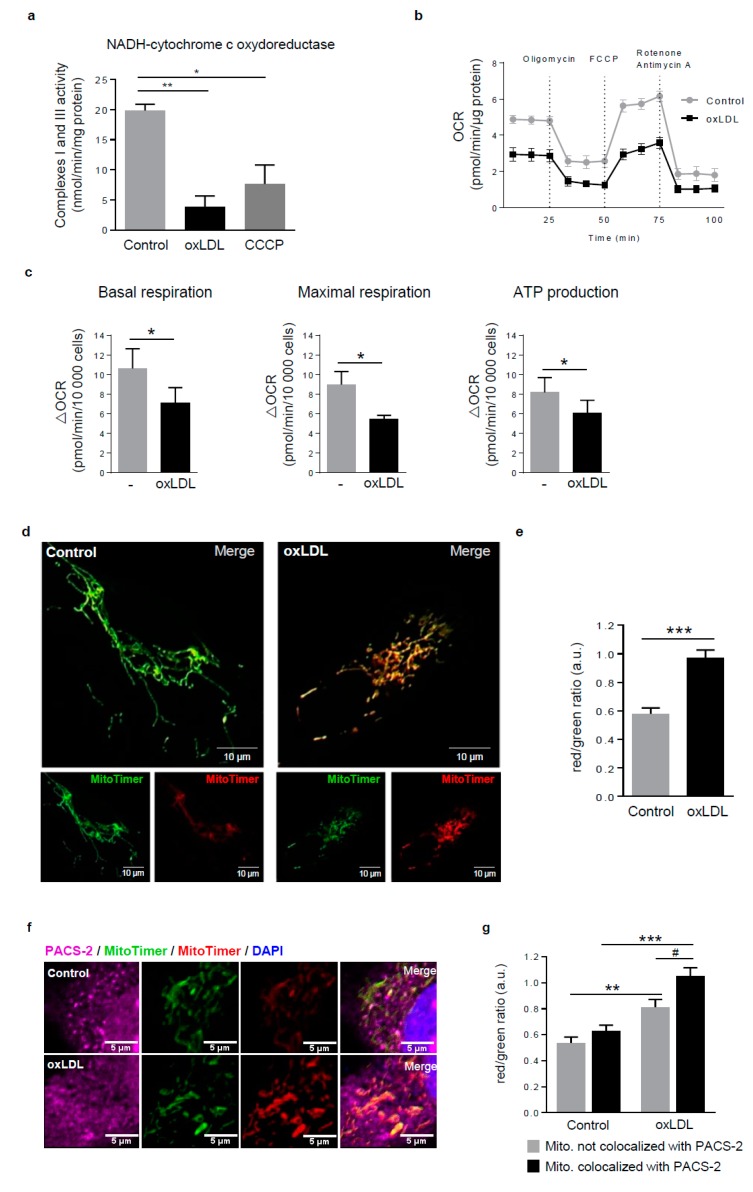
Dysfunctional mitochondria form MAM contact sites in response to oxidized LDL. (**a**) Respiratory chain complexes I+III activities were measured in hVSMCs treated or not (Control) with oxidized LDL (oxLDL, 200 µg ApoB/mL, 5 h) or carbonyl cyanide 3-chlorophenylhydrazone (CCCP) (20 µM). The graph represents the mean ± SEM of the absorbance at 550 nm of reduced cytochrome c in presence of NADH substrate (*n* = 4; Kruskall–Wallis test, * *p* < 0.05, ** *p* < 0.01). (**b**) Seahorse profile for oxygen consumption rate (OCR) in hVSMCs treated or not (Control) with oxidized LDL (oxLDL, 200 µg ApoB/mL, 5 h) following treatment with oligomycin, carbonyl cyanide 4-(trifluoromethoxy) phenylhydrazone (FCCP), and antimycin A/rotenone. Data are the mean ± SEM (*n* = 5). (**c**) Graphs represent the basal respiration (last rate measurement before first injection—non mitochondrial respiration rate), the maximal respiration (maximal rate measurement after FCCP injection—non mitochondrial respiration rate), and ATP production (last rate measurement before first injection—minimum rate measurement after oligomycin injection) in hVSMCs treated or not (Control) with oxidized LDL (oxLDL, 200 µg ApoB/mL, 5 h). Data are the mean ± SEM (*n* = 5; Wilcoxon test, * *p* < 0.05). (**d**) Imaging of MitoTimer, hVSMCs were transfected with a SIN-PGK-MitoTimer plasmid and exposed or not (Control) to oxidized LDL (oxLDL, 200 µg ApoB/mL, 5 h). Merged images of the green and red channels are shown; smaller insets show the green and red channels—scale bar 10 µm. (**e**) Red/green fluorescent ratio quantification of 10 cells analyzed per experiment for each condition (mean ± SEM; *n* = 3, Student’s *t* test, *** *p* < 0.001). (**f**) Representative images of PACS-2 (magenta) and MitoTimer (green and red) in hVSMCs at baseline conditions (Control) or stimulated with oxidized LDL (oxLDL, 200 µg ApoB/mL, 5 h). Images were obtained with an LSM 780 confocal microscope, scale bar 5 µm. (**g**) Red/green fluorescent ratio quantification was performed for mitochondria colocalized or not with PACS-2. Analyses were done with Image J software, and the graph represents the mean ± SEM of 10 cells analyzed per experiment for each condition (*n* = 3, two-way ANOVA with treatment (*) and mitochondria/PACS-2 colocalization (#) as category factors and Tukey’s post-hoc test, ** *p* < 0.01, *** *p* < 0.001, ^#^ *p* < 0.05).

We next asked whether the formation of MAMs induced by the treatment of hVSMCs with oxidized LDL is linked to a mitochondrial dysfunction. The specific activity of mitochondrial respiratory complexes I and III following treatment of hVSMCs with oxidized LDL was significantly impaired compared to untreated cells that used the mitochondrial oxidative phosphorylation uncoupler CCCP (Figure 3a). Mitochondrial bioenergetic functions were assessed using a Seahorse extracellular flux analyzer that measures the mitochondrial oxygen consumption rate (OCR) as an indicator of respiratory reserve capacity after uncoupling electron transport. Basal and maximal respiration, as well as ATP production, were each significantly reduced in oxidized LDL-stimulated hVSMCs compared to control conditions (Figure 3b,c). Since we recently identified the selective degradation of altered mitochondria by mitophagy as a safeguard mechanism against hVSMC apoptosis and because the site of mitophagosome biogenesis has been described at MAM contacts in yeast [22], we then investigated whether the altered mitochondria could be preferentially located at MAMs.

For that, we used the MitoTimer probe, a mitochondria-targeted green fluorescent protein which shifts irreversibly to red fluorescence when oxidized [28] in combination with the immunofluorescence detection of PACS-2. MitoTimer-transfected hVSMCs displayed at baseline a predominantly green mitochondrial network and the treatment with oxidized LDL caused an increase of the MitoTimer red fluorescence, corroborating the mitochondrial damage previously observed (Figure 3d). The fluorescence signal intensity in the red and green channels was determined at baseline and after oxidized LDL stimulation, allowing for the measurement of the red to green ratio, which was significantly increased in stimulated-hVSMCs compared to the normal condition (Figure 3e). The quantification of the colocalization between PACS-2 and each MitoTimer-positive mitochondrion revealed that, based on their higher red/green fluorescence ratio, oxidized LDL stimulated the association of PACS-2 with oxidized mitochondria. This result suggests that damaged mitochondria establish contacts with the ER to trigger mitophagosome formation (Figure 3f,g).

Consistent with this hypothesis, we showed that the ER protein oxidoreductase Ero1-Lα, which is involved in the regulation of redox processes [32], significantly re-localized to MAM contact sites under oxidized LDL stimulation (Figure S2a,b). Since we previously demonstrated that oxidized LDL induced an ER stress [24,33], our result suggests that Ero1-Lα could be involved in the control of the ER redox state to the formation of MAM contacts.

### 3.3. The Sorting Protein PACS-2 Is Necessary for the Process of Autophagosome Formation during Mitophagy in hVSMCs

**Figure 4:**
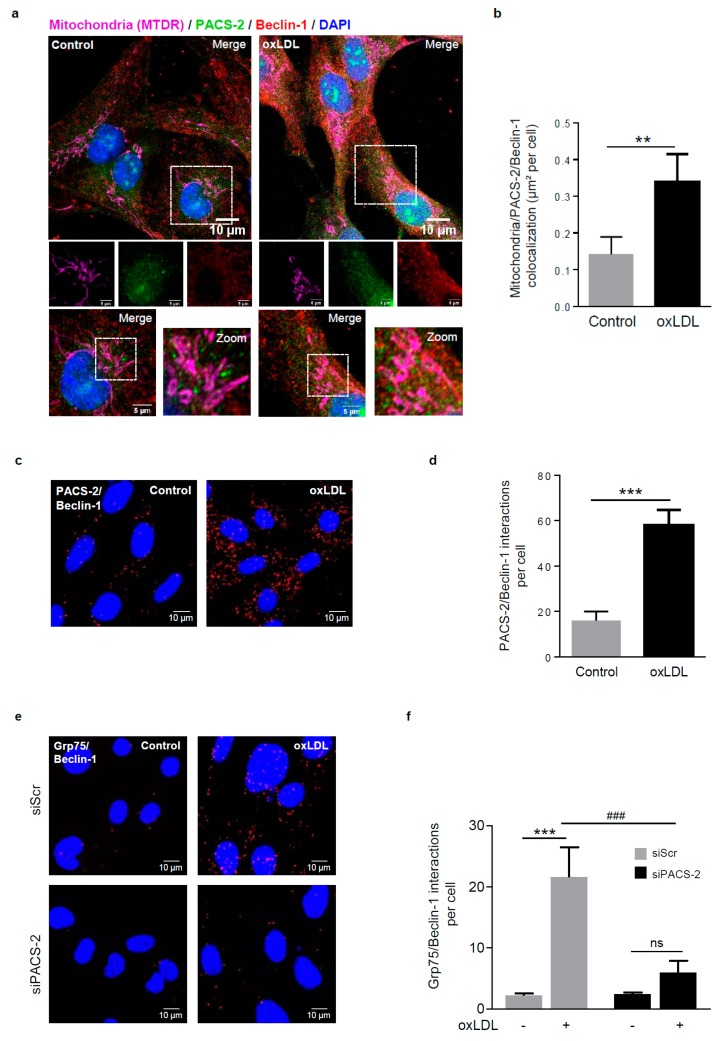
Autophagosome formation during mitophagy requires PACS-2 and MAM contact sites in hVSMCs. (**a**) Representative images of mitochondria (MTDR, magenta), PACS-2 (green) and Beclin-1 (red) in hVSMCs at baseline conditions (Control) or stimulated with oxidized LDL (oxLDL, 200 µg ApoB/mL, 5 h). Images were obtained with an LSM 780 confocal microscope, scale bar 10 µm, zoom scale bar 5 µm. (**b**) Analysis of the colocalization area between mitochondria, PACS-2, and Beclin-1 using Image J software. The graph represents the mean ± SEM of 10 cells analyzed per experiment for each condition (*n* = 3, Mann–Whitney test, ** *p* < 0.01). (**c**) Representative images of the interactions between PACS-2 and Beclin-1 (red dots) obtained by a proximity ligation assay (PLA) in hVSMCs stimulated or not (Control) with oxidized LDL (oxLDL, 200 µg ApoB/mL, 5 h). Images were obtained with an LSM 780 confocal microscope. (**d**) The number of PACS-2/Beclin-1 interactions per cell was analyzed using Image J software; the graph represents the mean ± SEM of five wide field images per experiment for each condition (*n* = 4; Mann–Whitney test, *** *p* < 0.001). (**e**) Representative images of the interactions between glucose-regulated protein 75 (GRP75) and Beclin-1 (red dots) obtained by proximity ligation assay (PLA). hVSMCs were transfected with scrambled siRNA (siScr) or siRNA PACS-2 (siPACS-2) and stimulated or not (Control) with oxidized LDL (oxLDL, 200 µg ApoB/mL, 5 h). Images were obtained with an LSM 780 confocal microscope. (**f**) The number of GRP75/Beclin-1 interactions per cell was analyzed using Image J software, the graph represents the mean ± SEM of 5 wide field images per experiment for each condition (*n* = 3; two-way ANOVA with treatment (*) and siRNA (#) as category factors and Tukey’s post-hoc test, ***p < 0.001, ### *p* < 0.001, ns, non-significant).

The MAM contact sites are crucial for mitochondria fission and the initiation of autophagosome formation following mitophagic stimuli [34]. To investigate whether pre-autophagosome markers are located at MAM sites upon oxidized LDL stimulation, we assessed, by confocal microscopy and PLA, the colocalization and the interaction between mitochondria, PACS-2, and the pre-autophagosomal marker Beclin-1. Triple-color imaging demonstrated, in stimulated-hVSMCs, a marked enhancement of Beclin-1 at MAM sites (Figure 4a,b), as well as an increased number of PLA dots (Figure 4c,d) compared to the baseline condition. To further substantiate this result, we quantified the fragmentation of mitochondria and the number of autophagosomes in oxidized LDL stimulated-hVSMCs. As expected, all these parameters were significantly increased (Figure S3a–c). Moreover, as we previously showed that the molecular mechanism mediating mitophagy in hVSMCs involved the recruitment of the E3 ubiquitin ligase Parkin to mitochondria, we also searched for the presence of Parkin. Indeed, PLA quantification showed the significant enhancement of VDAC1/Parkin and MFN2/Parkin interactions following oxidized LDL stimulation (Figure S3d,e). Interestingly, the increased interactions between Grp75 and Beclin-1 following oxidized LDL treatment were significantly prevented after PACS-2 silencing, as measured by PLA (Figure 4e,f), thus substantiating the major role of PACS-2 in the process of autophagosome formation during mitophagy.

### 3.4. The Absence of PACS-2 Enhances Cell Death and Impairs Mitophagy

**Figure 5:**
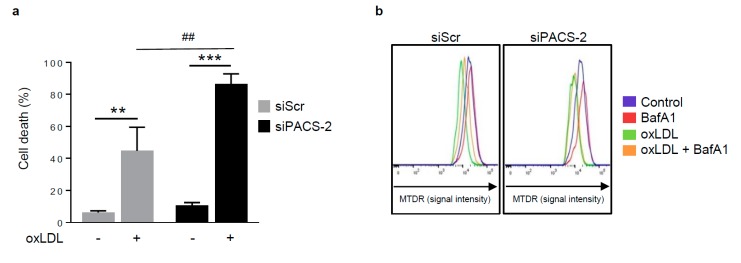

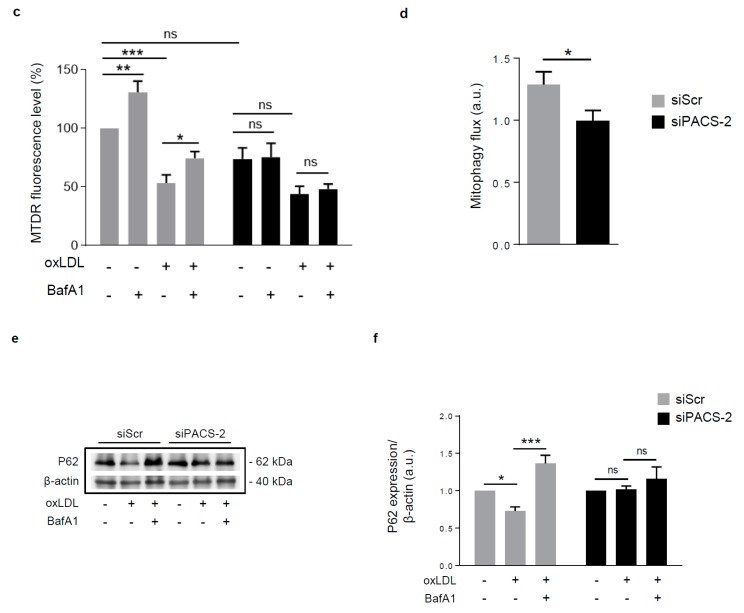

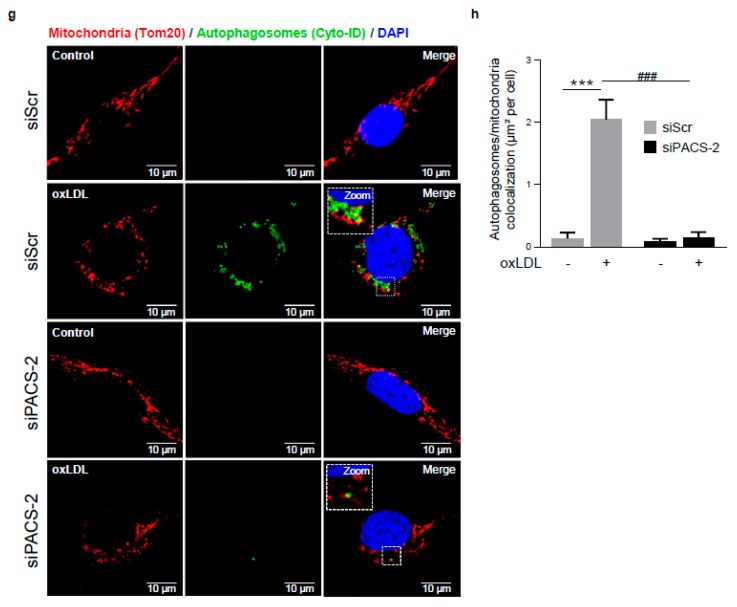
PACS-2 deletion enhances hVSMCs death and impairs mitophagy in response to oxidized LDL. (**a**) Cell death assessment by Annexin V/PI staining followed by flow cytometry analysis in hVSMCs transfected with scrambled siRNA (siScr) or siRNA PACS-2 (siPACS-2) and stimulated or not (Control) with oxidized LDL (oxLDL, 200 µg ApoB/mL, 8 h). The graph represents the quantitative analysis of the percentage of dead cells, data are expressed as mean ± SEM (*n* = 5–7; two-way ANOVA with treatment (*) and siRNA (#) as category factors and Tukey’s post-hoc test, ** *p* < 0.01, *** *p* < 0.001, ^##^ *p* < 0.01). (**b**) Representative overlays of mitophagy analysis determined by flow cytometry using the MTDR staining. Cells were transfected with scrambled siRNA (siScr) or siRNA PACS-2 (siPACS-2) and stimulated or not (Control) with oxidized LDL (oxLDL, 200 µg ApoB/mL, 8 h). Cells were treated with or without bafilomycin A1 (BafA1, 100 nM) before analysis to block lysosomal degradation and stained with MTDR for flow cytometry analysis. (**c**) Mitophagy assessment in hVSMCs. The graph represents the quantitative analysis of the percentage of MTDR fluorescence; data are expressed as mean ± SEM (*n* = 5–9; two-way ANOVA with treatment (*) and siRNA (#) as category factors and Holm–Sidak’s post-hoc test, * *p* < 0.05, ** *p* < 0.01, *** *p* < 0.001, ns, non-significant). (**d**) Mitophagy flux determination (arbitrary unit, a.u.) in hVSMCs transfected with scrambled siRNA (siScr) or siRNA PACS-2 (siPACS-2) and stimulated with oxidized LDL (oxLDL, 200 µg ApoB/mL, 8 h) in the presence of bafilomycin A1 (Baf1, 100 nM). Data are expressed as mean ± SEM (*n* = 5–9; Student’s *t* test, * *p* < 0.05). (**e**) Western-blot analysis of P62 expression in hVSMCs transfected with scrambled siRNA (siScr) or siRNA PACS-2 (siPACS-2) and stimulated or not (Control) with oxidized LDL (oxLDL, 200 µg ApoB/mL, 8 h) in the presence of bafilomycin A1 (Baf1, 100 nM). β-actin was used as the loading control. (**f**) The graph represents the densitometric analysis of the expression level of P62. The data are expressed as mean ± SEM of six independent experiments (one-way ANOVA and Holm–Sidak’s post-hoc test, * *p* < 0.05, *** *p* < 0.001, ns, non-significant). (**g**) Representative images of mitochondria (Tom20, red), autophagosomes (Cyto-ID, green) and nucleus (DAPI, blue) in hVSMCs transfected with scrambled siRNA (siScr) or siRNA PACS-2 (siPACS-2) and stimulated or not (Control) with oxidized LDL (oxLDL, 200 µg ApoB/mL, 8 h). Images were obtained with an LSM 780 confocal microscope, scale bar 10 µm. (**h**) Analysis of the colocalization area between autophagosomes and mitochondria using Image J software. The graph represents the mean ± SEM of 10 cells analyzed per experiment for each condition (*n* = 3, two-way ANOVA with treatment (*) and siRNA (#) as category factors and Holm–Sidak’s post-hoc test, *** *p* < 0.001, ^###^ *p* < 0.001).

We next investigated the outcomes associated with the absence of PACS-2 in hVSMCs in stress conditions. The consequences on cell fate were illustrated by the significant increased percentage of cell death in PACS-2-deficient hVSMCs following oxidized LDL treatment, compared to scramble siRNA transfected cells (Figure 5a). We then asked whether the increase of cell death could be a consequence of impaired mitophagy. For this purpose, we used a recently validated quantitative method to measure mitophagic flux by flow cytometry in the presence of lysosomal inhibitors [23,29]. As shown, the oxidized LDL treatment of hVSMCs reduced MTDR staining indicating increased mitochondria autophagy, which was prevented by the treatment with the lysosomal inhibitor bafilomycin A1 (BafA1). Notably, the MTDR signal measured in PACS-2-deficient hVSMCs treated with or without oxidized LDL was not significantly different (Figure 5b,c). In addition, the mitophagy flux, defined as the ratio of MTDR fluorescence in the presence of lysosomal inhibitors to that in the absence of inhibitors (normalized to the corresponding value in non oxLDL-treated cells), was significantly decreased in PACS-2-deleted hVSMCs, thus confirming that PACS-2 expression was necessary for the mitophagy process (Figure 5d). We also checked that the treatment with BafA1 did not interfere with upstream mitophagy pathway. Accordingly, we still measured an increase in mitochondria-ER contacts in BafA1 stimulated-hVSMCs following oxidized LDL treatment as well as mitochondria fragmentation (Figure S4a–c). Moreover, an analysis of autophagic flux by measuring P62 degradation in the presence of BafA1 or autophagosomes and mitochondria colocalization clearly supports the functional role of PACS-2 during autophagosome biogenesis (Figure 5e–h).

**Figure 6:**
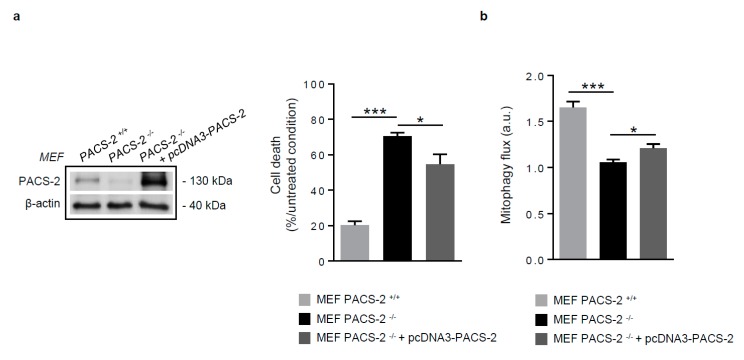
PACS-2 rescue in PACS-2-deficient cells prevents cell death and restores mitophagy following oxidized LDL treatment. (**a**) Cell death assessment by Annexin V/PI staining followed by flow cytometry analysis, in mouse embryonic fibroblasts (MEF) PACS-2 ^+/+^, MEF PACS-2 ^−/−^, and MEF PACS-2 ^−/−^ transfected with pcDNA3-PACS-2 stimulated with oxidized LDL (oxLDL, 200 µg ApoB/mL, 8 h). Data are expressed as mean ± SEM (*n* = 3; one-way ANOVA and Holm–Sidak’s post-hoc test, * *p* < 0.05, *** *p* < 0.001). (**b**) Mitophagy flux determination (arbitrary unit, a.u.) in MEF PACS-2 ^+/+^, MEF PACS-2 ^−/−^, and MEF PACS-2 ^−/−^ transfected with pcDNA3-PACS-2 and stimulated with oxidized LDL (oxLDL, 200 µg ApoB/mL, 8 h) in the presence of bafilomycin A1 (Baf1, 100 nM). Data are expressed as mean ± SEM (*n* = 6; one-way ANOVA and Holm–Sidak’s post-hoc test, * *p* < 0.05, *** *p* < 0.001).

We then hypothesized that the re-expression of PACS-2 would reverse the impairment of mitophagy and decrease cell death. For that, mouse embryonic fibroblasts (MEF) obtained from mice genetically deleted for PACS-2 (MEF PACS-2 ^−/−^) were transfected with a PACS-2 expression vector and stimulated with oxidized LDL. As shown, cell death was prevented, and mitophagy flux was recovered when cells re-express PACS-2, thus providing clear evidence on the new role of PACS-2 in the regulation of mitophagosome formation and cell fate (Figure 6a,b). Altogether, these findings suggest that PACS-2, which promotes MAM tethering, is a key regulator of autophagosome formation following mitophagic stimuli.

## 4. Discussion

Despite recent evidence underlining the importance of mitochondria-ER contact sites in cardiac tissue physiology, little is known about MAM communication and function in vascular tissues, including in VSMCs. Whereas mitochondrial dynamics through MFN2 expression have been described to play a role in VSMC phenotypic switching [35,36], there had been no direct or indirect evidence about a function of mitochondria-ER contacts in VSMCs. Our data show for the first time that the sorting protein PACS-2 regulates mitochondria-ER contact formation in human VSMCs stressed with oxidized lipoproteins. We also provide compelling evidence that PACS-2, as a MAM-related protein, participates in the mitophagy process in response to oxidized lipoproteins.

**Figure 7:**
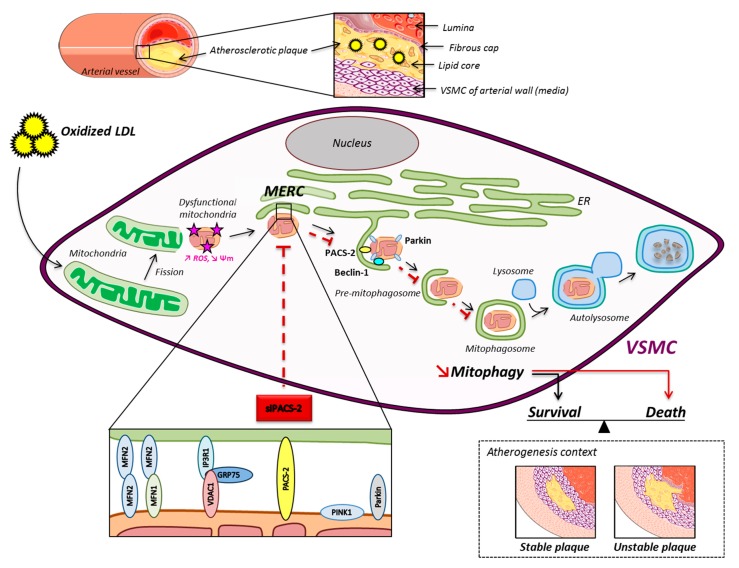
Schematic overview of the oxidized LDL effects on mitochondria and ER contacts in hVSMCs. The mitochondrial network fragments upon oxidized LDL treatment, and damaged mitochondria interact with the ER to form MAM contact sites. These subcellular platforms are increased in the atherogenic condition, and PACS-2 is important for the tethering. The pro-autophagic proteins Beclin-1 and Parkin are recruited in MAM contacts to promote the formation of the phagosomal membranes. The degradation of altered mitochondria by the mitophagic process promotes the survival of VSMC, leading to the stability of the atherosclerotic plaque.

Our previous works demonstrated that the successful autophagy of dysfunctional mitochondria stimulates VSMC survival against pro-apoptotic oxidized lipoproteins [23], whereas defective autophagy promotes VSMC apoptosis and unstable atherosclerotic plaque phenotype [14]. Interestingly, mitochondrial-ER contacts contribute to general autophagy in mammalian cells through the cooperation of the organelles that provide lipids for the growth of the isolation membrane [7,8]. However, although efficient mitophagy in yeast depends on the coupling of mitochondria and the ER through an ERMES complex which colocalizes with sites of mitophagosome biogenesis [22], it is currently unknown whether an analogous mechanism exists in mammalian cells. The use of high-resolution fluorescence methods (STED confocal microscopy and proximity ligation assay) allowed us for the first time: i) To measure an increase in mitochondria-ER contacts in stressed hVSMCs, and ii) to show that PACS-2 is necessary to establish those organelle contacts. Indeed, the disruption of ER-mitochondrial contacts by knocking down PACS-2 impairs mitophagosome formation and favors oxidized lipoproteins-induced cell apoptosis, thus supporting the hypothesis that MAM integrity is a prerequisite for mitochondria autophagy in mammalian cells. Moreover, our results are consistent with the work of Hamasaki [8], which showed that PACS-2 knockdown abolished autophagosme formation. To go further into the significance of mitochondria-ER contact formation in hVSMCs, we provide evidence that mitochondria turned dysfunctional after being challenged with oxidized lipoproteins. Indeed, we measured an increase of fragmented mitochondria with reduced bioenergetic efficiency and more oxidative stress in agreement with our previous results [14]. Consistently, the ER oxidoreductase Ero1-Lα colocalized with PACS-2 and mitochondria, and it also increased a shift to red fluorescence of a MitoTimer probe upon oxidized lipoproteins stimulation. Thus, we hypothesize that oxidized dysfunctional mitochondria behave as an attractive signal for the ER, resulting in the formation of MAM contacts. As alluded to previously, mitochondria-ER contact sites control cell function, including autophagy activity. Here, we describe a novel pathway where omegasomes form at MAMs in situation of oxidized lipoproteins-induced mitophagy; in addition, Beclin-1 and PACS-2 are involved in this process. Interestingly, an enrichment of Beclin-1 and PINK1 in the MAM compartment was recently described in SH-SY5Y cells stably expressing PINK1 and treated with CCCP [34]. Therefore, it is tempting to speculate that mitochondria-residing Beclin-1 and the ER protein PACS-2 interact to promote inter-organellear tethering and initiate the formation of the mitophagosome. In addition, the E3 ubiquitin ligase Parkin has been shown to accumulate at MAMs following glutamate excitotoxicity and to modulate mitochondria-ER communication [37,38]. We previously demonstrated that the molecular mechanism mediating mitophagy in human VSMC involved the recruitment of PINK1 and Parkin to mitochondria [23]. Our new results showed the interaction of Parkin with MAM-residing proteins. Consequently, we can propose the following scheme (Figure 7, scheme) where, through inter-organelle communication, dysfunctional mitochondria may be sensed by MAMs and be followed by mitophagy induction and removal, thus avoiding cell death and favoring cell survival.

Finally, our findings firstly uncover new insights on the significance of mitochondria-ER contact sites in cell vascular physiology and a disease context which has been poorly investigated as a critical broad-spectrum mechanism. Secondly, manipulating mitochondria-ER communication can open new opportunities for future research, leading to the development of more specific strategies to selectively improve VSMC survival and stabilize atherosclerotic plaques.

## Supplementary Materials

The following are available online at https://www.mdpi.com/2073-4409/8/6/638/s1, Figure S1, S2, S3 and S4.

## References

[1] Vance J.E. (2014). MAM (mitochondria-associated membranes) in mammalian cells: Lipids and beyond. Biochim. Biophys. Acta.

[2] Lang A., John Peter A.T., Kornmann B. (2015). ER-mitochondria contact sites in yeast: Beyond the myths of ERMES. Curr. Opin. Cell Biol..

[3] Filadi R., Theurey P., Pizzo P. (2017). The endoplasmic reticulum-mitochondria coupling in health and disease: Molecules, functions and significance. Cell Calcium.

[4] Naon D., Scorrano L. (2014). At the right distance: ER-mitochondria juxtaposition in cell life and death. Biochim. Biophys. Acta.

[5] Prudent J., McBride H.M. (2017). The mitochondria-endoplasmic reticulum contact sites: A signalling platform for cell death. Curr. Opin. Cell Biol..

[6] van Vliet A.R., Verfaillie T., Agostinis P. (2014). New functions of mitochondria associated membranes in cellular signaling. Biochim. Biophys. Acta.

[7] Hailey D.W., Rambold A.S., Satpute-Krishnan P., Mitra K., Sougrat R., Kim P.K., Lippincott-Schwartz J. (2010). Mitochondria supply membranes for autophagosome biogenesis during starvation. Cell.

[8] Hamasaki M., Furuta N., Matsuda A., Nezu A., Yamamoto A., Fujita N., Oomori H., Noda T., Haraguchi T., Hiraoka Y. (2013). Autophagosomes form at ER-mitochondria contact sites. Nature.

[9] Simmen T., Aslan J.E., Blagoveshchenskaya A.D., Thomas L., Wan L., Xiang Y., Feliciangeli S.F., Hung C.H., Crump C.M., Thomas G. (2005). PACS-2 controls endoplasmic reticulum-mitochondria communication and Bid-mediated apoptosis. EMBO J..

[10] Barroso-Gonzalez J., Auclair S., Luan S., Thomas L., Atkins K.M., Aslan J.E., Thomas L.L., Zhao J., Zhao Y., Thomas G. (2016). PACS-2 mediates the ATM and NF-kappaB-dependent induction of anti-apoptotic Bcl-xL in response to DNA damage. Cell Death Differ..

[11] Aslan J.E., You H., Williamson D.M., Endig J., Youker R.T., Thomas L., Shu H., Du Y., Milewski R.L., Brush M.H. (2009). Akt and 14-3-3 control a PACS-2 homeostatic switch that integrates membrane traffic with TRAIL-induced apoptosis. Mol. Cell.

[12] Clarke M.C., Figg N., Maguire J.J., Davenport A.P., Goddard M., Littlewood T.D., Bennett M.R. (2006). Apoptosis of vascular smooth muscle cells induces features of plaque vulnerability in atherosclerosis. Nat. Med..

[13] Vindis C. (2015). Autophagy: An emerging therapeutic target in vascular diseases. Br. J. Pharm..

[14] Nahapetyan H., Moulis M., Grousset E., Faccini J., Grazide M.H., Mucher E., Elbaz M., Martinet W., Vindis C. (2019). Altered mitochondrial quality control in Atg7-deficient VSMCs promotes enhanced apoptosis and is linked to unstable atherosclerotic plaque phenotype. Cell Death Dis..

[15] Klionsky D.J., Emr S.D. (2000). Autophagy as a regulated pathway of cellular degradation. Science.

[16] Lemasters J.J. (2005). Selective mitochondrial autophagy, or mitophagy, as a targeted defense against oxidative stress, mitochondrial dysfunction, and aging. Rejuvenation Res..

[17] Mortensen M., Ferguson D.J., Simon A.K. (2010). Mitochondrial clearance by autophagy in developing erythrocytes: Clearly important, but just how much so?. Cell Cycle.

[18] Kubli D.A., Gustafsson A.B. (2012). Mitochondria and mitophagy: The yin and yang of cell death control. Circ. Res..

[19] Springer W., Kahle P.J. (2011). Regulation of PINK1-Parkin-mediated mitophagy. Autophagy.

[20] Karbowski M., Youle R.J. (2003). Dynamics of mitochondrial morphology in healthy cells and during apoptosis. Cell Death Differ..

[21] Galluzzi L., Kepp O., Trojel-Hansen C., Kroemer G. (2012). Mitochondrial control of cellular life, stress, and death. Circ. Res..

[22] Bockler S., Westermann B. (2014). Mitochondrial ER contacts are crucial for mitophagy in yeast. Dev. Cell.

[23] Swiader A., Nahapetyan H., Faccini J., D’Angelo R., Mucher E., Elbaz M., Boya P., Vindis C. (2016). Mitophagy acts as a safeguard mechanism against human vascular smooth muscle cell apoptosis induced by atherogenic lipids. Oncotarget.

[24] Larroque-Cardoso P., Swiader A., Ingueneau C., Negre-Salvayre A., Elbaz M., Reyland M.E., Salvayre R., Vindis C. (2013). Role of protein kinase C delta in ER stress and apoptosis induced by oxidized LDL in human vascular smooth muscle cells. Cell Death Dis..

[25] Vindis C., Elbaz M., Escargueil-Blanc I., Auge N., Heniquez A., Thiers J.C., Negre-Salvayre A., Salvayre R. (2005). Two distinct calcium-dependent mitochondrial pathways are involved in oxidized LDL-induced apoptosis. Arter. Thromb. Vasc. Biol..

[26] Spinazzi M., Casarin A., Pertegato V., Salviati L., Angelini C. (2012). Assessment of mitochondrial respiratory chain enzymatic activities on tissues and cultured cells. Nat. Protoc..

[27] van der Windt G.J., Chang C.H., Pearce E.L. (2016). Measuring Bioenergetics in T Cells Using a Seahorse Extracellular Flux Analyzer. Curr. Protoc. Immunol..

[28] Hernandez G., Thornton C., Stotland A., Lui D., Sin J., Ramil J., Magee N., Andres A., Quarato G., Carreira R.S. (2013). MitoTimer: A novel tool for monitoring mitochondrial turnover. Autophagy.

[29] Mauro-Lizcano M., Esteban-Martinez L., Seco E., Serrano-Puebla A., Garcia-Ledo L., Figueiredo-Pereira C., Vieira H.L., Boya P. (2015). New method to assess mitophagy flux by flow cytometry. Autophagy.

[30] Thomas G., Aslan J.E., Thomas L., Shinde P., Shinde U., Simmen T. (2017). Caught in the act-protein adaptation and the expanding roles of the PACS proteins in tissue homeostasis and disease. J. Cell Sci..

[31] Paillard M., Tubbs E., Thiebaut P.A., Gomez L., Fauconnier J., Da Silva C.C., Teixeira G., Mewton N., Belaidi E., Durand A. (2013). Depressing mitochondria-reticulum interactions protects cardiomyocytes from lethal hypoxia-reoxygenation injury. Circulation.

[32] Simmen T., Lynes E.M., Gesson K., Thomas G. (2010). Oxidative protein folding in the endoplasmic reticulum: Tight links to the mitochondria-associated membrane (MAM). Biochim. Biophys. Acta.

[33] Muller C., Salvayre R., Negre-Salvayre A., Vindis C. (2011). HDLs inhibit endoplasmic reticulum stress and autophagic response induced by oxidized LDLs. Cell Death Differ.

[34] Gelmetti V., De Rosa P., Torosantucci L., Marini E.S., Romagnoli A., Di Rienzo M., Arena G., Vignone D., Fimia G.M., Valente E.M. (2017). PINK1 and BECN1 relocalize at mitochondria-associated membranes during mitophagy and promote ER-mitochondria tethering and autophagosome formation. Autophagy.

[35] Guo X., Chen K.H., Guo Y., Liao H., Tang J., Xiao R.P. (2007). Mitofusin 2 triggers vascular smooth muscle cell apoptosis via mitochondrial death pathway. Circ. Res..

[36] Morales P.E., Torres G., Sotomayor-Flores C., Pena-Oyarzun D., Rivera-Mejias P., Paredes F., Chiong M. (2014). GLP-1 promotes mitochondrial metabolism in vascular smooth muscle cells by enhancing endoplasmic reticulum-mitochondria coupling. Biochem. Biophys. Res. Commun..

[37] Cali T., Ottolini D., Negro A., Brini M. (2013). Enhanced parkin levels favor ER-mitochondria crosstalk and guarantee Ca(2+) transfer to sustain cell bioenergetics. Biochim. Et Biophys. Acta.

[38] Van Laar V.S., Roy N., Liu A., Rajprohat S., Arnold B., Dukes A.A., Holbein C.D., Berman S.B. (2015). Glutamate excitotoxicity in neurons triggers mitochondrial and endoplasmic reticulum accumulation of Parkin, and, in the presence of N-acetyl cysteine, mitophagy. Neurobiol. Dis..

