# Supplementary material for: The Multifunctional Sorting Protein PACS-2 Controls Mitophagosome Formation in Human Vascular Smooth Muscle Cells through Mitochondria-ER Contact Sites
Source: Cells. 2019 Jun 25;8(6):638. doi: 10.3390/cells8060638 (PMC6627983; doi:10.3390/cells8060638)
Supplement: Supplementary file 1 [file cells-08-00638-s001.pdf]

Figure S1

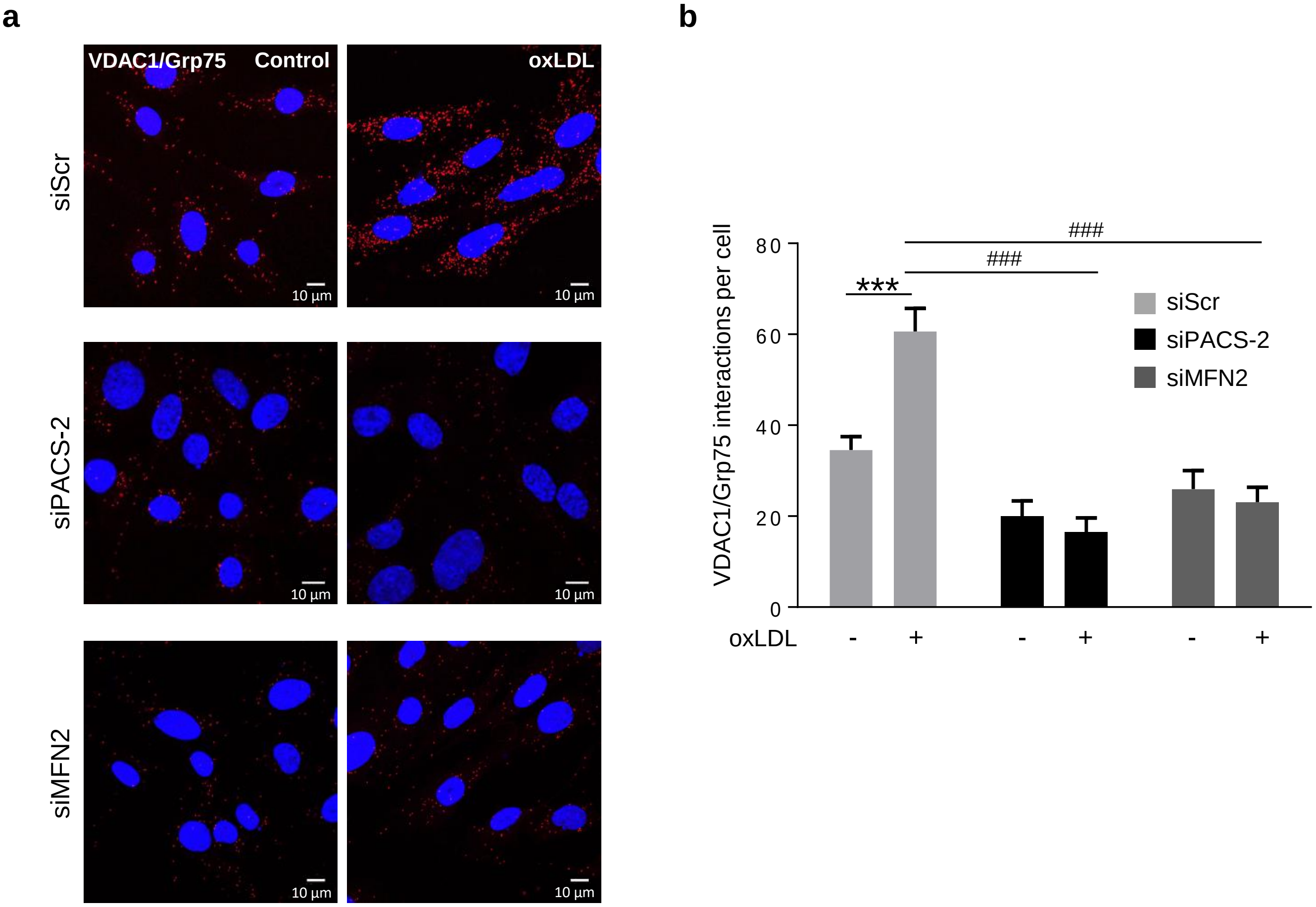

a, Representative images of the interactions between the two proteins VDAC1 and Grp75 visualized as red dots. hVSMCs were transfected with scrambled siRNA (siScr), PACS-2 siRNA (siPACS-2) or MFN2 siRNA (siMFN2) and stimulated or not with oxLDL (200 µg ApoB/mL, 5h). Images were obtained with a LSM 780 confocal microscope. **b,** The number of VDAC1/Grp75 interactions per cell was analyzed with Image J software using 5 to 10 wide field images per experiment for each condition (mean ± SEM; n=3; two-way ANOVA with treatment (*) and siRNA (#) as category factors and Tukey's post-hoc test, *** ### $p<0.001$).

Figure S2

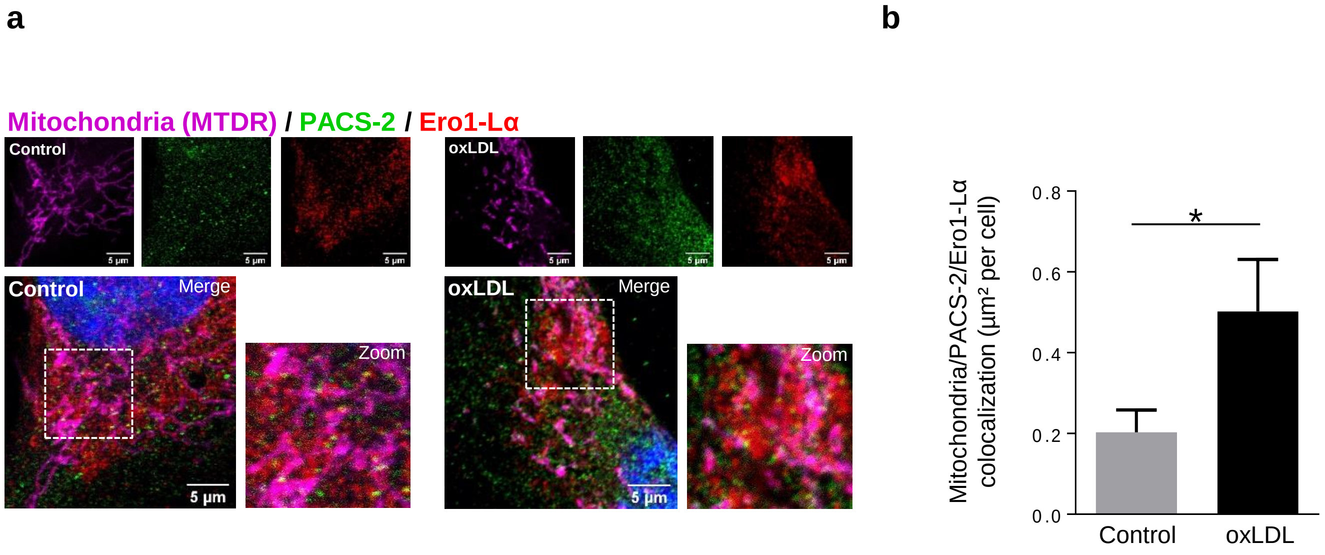

a, Representative images of the colocalization between mitochondria (MTDR, magenta), PACS-2 (green) and Ero1-Lα (red) in hVSMCs treated with oxLDL (200 µg ApoB/mL, 5h) or not (Control). Images were obtained with a LSM780 confocal microscope. **b,** Analysis of the colocalization area between MAM (mitochondria-PACS-2 colocalization) and Ero1-Lα with Image J software using 10 images of one cell per experiment for each condition (mean ± SEM; n=3; Mann-Whitney test, *p<0.05).

Figure S3

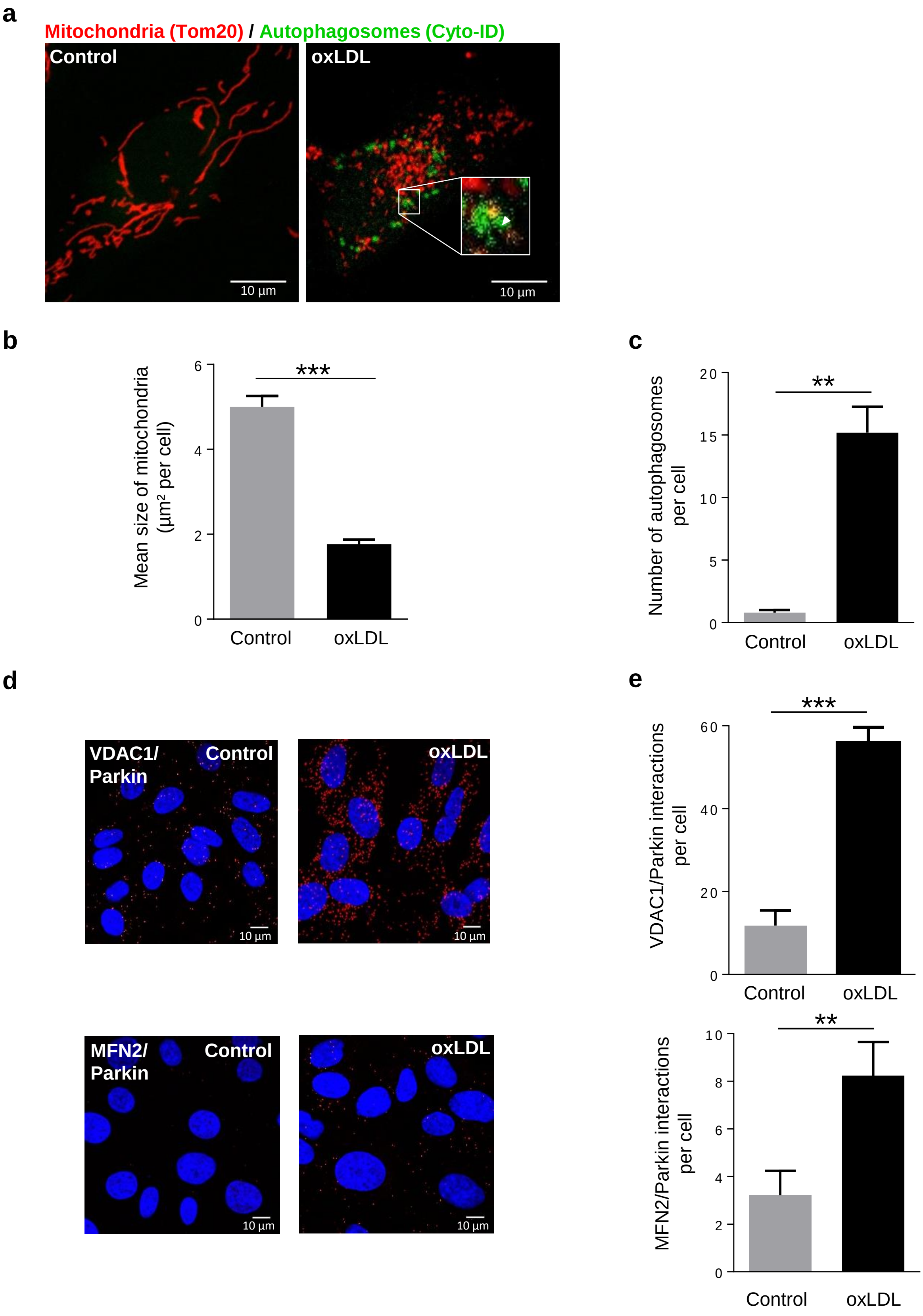

a, Representative images of the mitochondrial network (Tom20, red) and autophagosomes (Cyto-ID, green) in hVSMCs treated with oxLDL (200 µg ApoB/mL, 8h) or at baseline conditions (Control). Images were obtained with a LSM780 confocal microscope and the enlarged box in oxLDL condition shows a mitophagic event (colocalization between an autophagosome and a mitochondrion). **b,** Analysis of the mean area of mitochondria per cell with Image J software using 10 images of one cell per experiment for each condition (mean ± SEM; n=3; Mann-Whitney test, ***$p<0.001$). **c,** Analysis of the number of autophagosomes per cell with Image J software using 5 images of one cell per experiment for each condition (mean ± SEM; Mann-Whitney test, **$p<0.01$). **d,** Representative images of the VDAC1/Parkin and MFN2/Parkin interactions as red dots, in hVSMCs stimulated or not (Control) with oxLDL (200 µg ApoB/mL, 8h). Images were obtained with a LSM 780 confocal microscope. **e,** The number of VDAC1/Parkin and MFN2/Parkin interactions per cell was analyzed with Image J software using respectively 10 and 5 wide field images per experiment for each condition (mean ± SEM; n=3; Mann-Whitney test, **$p<0.01$, ***$p<0.001$).

Figure S4

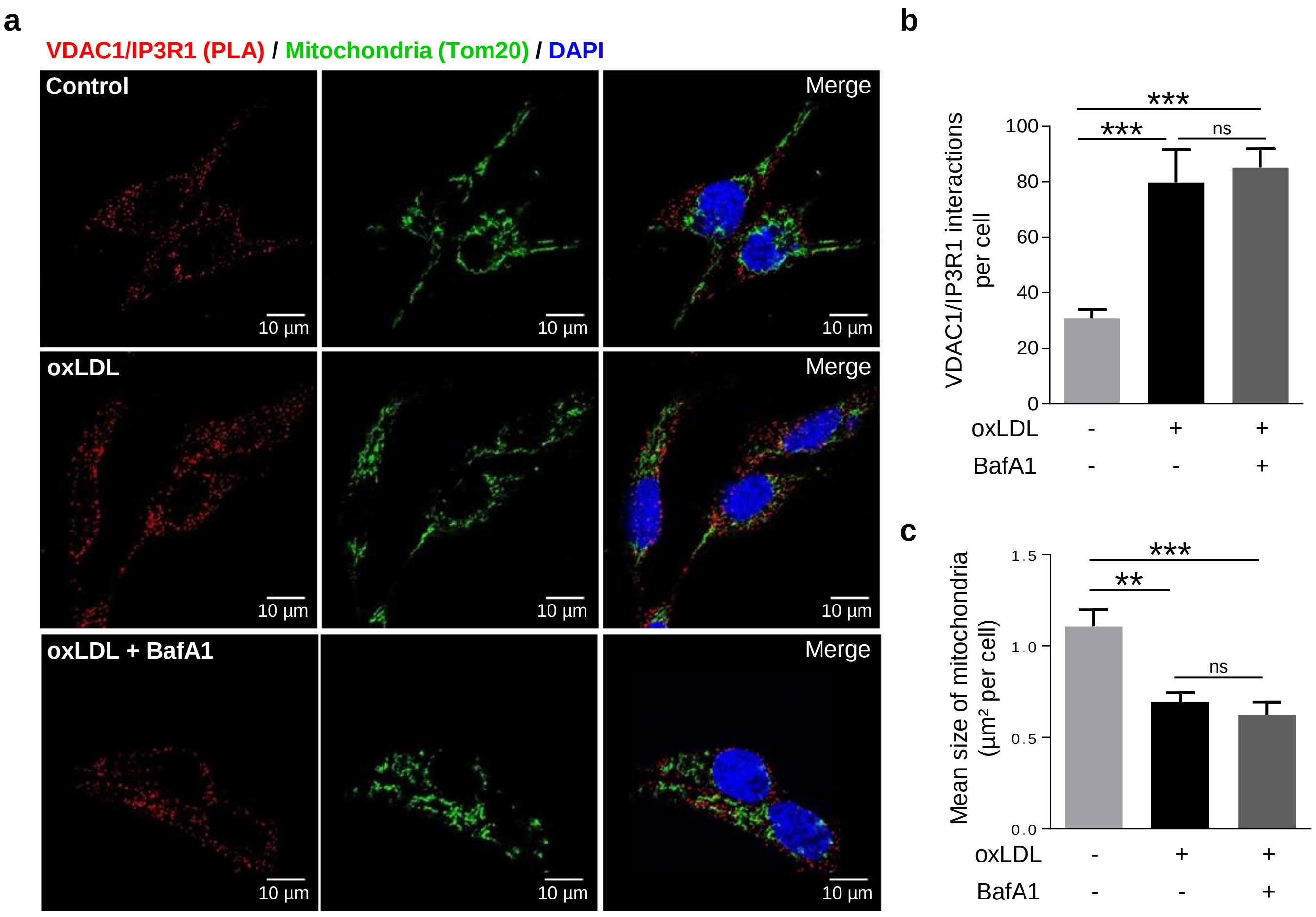

a, Representative images of the interactions between VDAC1 and IP3R1 (red dots) obtained by PLA and the labelled mitochondrial network (Tom20, green) in hVSMCs stimulated or not (Control) with oxidized LDL (oxLDL, 200 µg ApoB/mL, 5h) in the presence of bafilomycin A1 (BafA1, 100 nM). Images were obtained with a LSM 780 confocal microscope. **b,** The number of VDAC1/IP3R1 interactions per cell was analyzed using Image J software, the graph represents the mean ± SEM of 5 wide field images per experiment for each condition (n=2; one-way ANOVA test, *** $p<0.001$, ns, non-significant). **c,** Analysis of the mean area of mitochondria per cell with Image J software using 10 images per experiment for each condition (mean ± SEM; n=2; one-way ANOVA test, ** $p<0.01$, *** $p<0.001$, ns, non-significant).
